
JOURNAL INFORMATION
==============================
NLM Title Abbreviation: Wirtschaftsdienst
Journal ID: Wirtschaftsdienst
NLM Journal ID: 101086612

Wirtschaftsdienst (Hamburg, Germany : 1949)

ISSN: 0043-6275

ARTICLE INFORMATION
==============================
PMCID: PMC7368612
PMID: 32834165
DOI: 10.1007/s10273-020-2692-5
Article ID: 2692
Article version: 1
Subjects: Zeitgespräch

Konjunkturpaket notwendig — Rückkehr zur Schuldenbremse nicht forcieren

Gechert Sebastian Sebastian-Gechert@BOECKLER.DE
1
Paetz Christoph Christoph-Paetz@BOECKLER.DE
1
Truger Achim achim.truger@uni-due.de
2
1 in der Hans-Böckler-Stiftung: Makroökonomie und Einkommensverteilung, Institut für Makroökonomie und Konjunkturforschung (IMK), Georg-Glock-Str. 18, 40474 Düsseldorf, Deutschland
2 grid.5718.b 0000 0001 2187 5445 Institut für Sozioökonomie, Universität Duisburg-Essen, Forsthausweg 2, 47057 Duisburg, Deutschland
Electronic publication date: 2020 Jul 18
Print publication date: 2020
Volume: 100
Issue: 7
First page: 493
Last page: 497
Copyright: © Der/die Autor(en) 2020
License: Open Access Dieser Artikel wird unter der Creative Commons Namensnennung 4.0 International Lizenz veröffentlicht, welche die Nutzung, Vervielfältigung, Bearbeitung, Verbreitung und Wiedergabe in jeglichem Medium und Format erlaubt, sofern Sie den/die ursprünglichen Autor(en) und die Quelle ordnungsgemäß nennen, einen Link zur Creative Commons Lizenz beifügen und angeben, ob Änderungen vorgenommen wurden.
Die in diesem Artikel enthaltenen Bilder und sonstiges Drittmaterial unterliegen ebenfalls der genannten Creative Commons Lizenz, sofern sich aus der Abbildungslegende nichts anderes ergibt. Sofern das betreffende Material nicht unter der genannten Creative Commons Lizenz steht und die betreffende Handlung nicht nach gesetzlichen Vorschriften erlaubt ist, ist für die oben aufgeführten Weiterverwendungen des Materials die Einwilligung des jeweiligen Rechteinhabers einzuholen.
Weitere Details zur Lizenz entnehmen Sie bitte der Lizenzinformation auf http://creativecommons.org/licenses/by/4.0/deed.de.
License URL: https://creativecommons.org/licenses/by/4.0/

issue-copyright-statement: © The Author(s) 2020

==============================
Dr. Sebastian Gechert ist Referatsleiter für Makroökonomie und Einkommensentwicklung am Institut für Makroökonomie und Konjunkturforschung (IMK) der Hans-Böckler-Stiftung.

Christoph Paetz ist wissenschaftlicher Mitarbeiter am IMK und Doktorand an der Universität Duisburg-Essen.

Prof. Dr. Achim Truger ist Professor für Sozioökonomie mit dem Schwerpunkt Staatstätigkeit und Staatsfinanzen am Institut für Sozioökonomie an der Universität Duisburg-Essen, Mitglied des Sachverständigenrats zur Begutachtung der gesamtwirtschaftlichen Entwicklung und Senior Research Fellow am IMK.

Die Corona-Krise hat zu einer tiefen globalen Rezession geführt. Das deutsche reale Bruttoinlandsprodukt (BIP) wird im Jahr 2020 in historischem Ausmaß um voraussichtlich mehr als 6 % gegenüber dem Vorjahr zurückgehen. Die Bundesregierung hat seit März 2020 sehr schnell großvolumige Maßnahmen zur Überbrückung und zur Abmilderung der wirtschaftlichen Folgen der Krise und der notwendigen behördlichen Einschränkungen ergriffen. Nach der begonnenen Lockerung hat sie nun ein Konjunkturpaket im Umfang von 130 Mrd. Euro aufgelegt, um die wirtschaftliche Erholung zu beschleunigen.

te bei Arbeitslosigkeit und Kapitalstock zu vermeiden. Im Vergleich zur Finanzkrise 2008/2009 wurde dieses Mal nicht erst lange gezögert und eine Grundsatzdebatte geführt, sondern unverzüglich gehandelt. Vor der Finanzkrise spielte diskretionäre Fiskalpolitik tendenziell eine untergeordnete Rolle bei der Stabilisierung der Wirtschaft. Sie stellte auch keinen großen Schwerpunkt in der ökonomischen Forschung mehr dar. Das hat sich in den vergangenen Jahren drastisch geändert (Furman, 2016). Demnach kann die Fiskalpolitik sich mit guten Erfolgsaussichten an den sogenannten drei Ts als Kriterien für wirksame konjunkturstützende Maßnahmen orientieren: Die Finanzpolitik sollte „timely, targeted und temporary“ (Elmendorf und Furman, 2008) sein, also rechtzeitig, gezielt und befristet eingesetzt werden. Verschiedene fiskalpolitische Instrumente unterscheiden sich in ihrer Effektivität, aber grundsätzlich gilt, dass aktive Konjunkturpolitik im Sinne der drei Ts wirksam ist.

In herkömmlichen Makromodellen zeigt sich, dass transitorische fiskalpolitische Maßnahmen Multiplikatoreffekte bis über 2 erreichen können, wenn sich die Geldpolitik am sogenannten Zero Lower Bound befindet (Christiano et al., 2011; Woodford, 2011). Der Befund hoher Wirksamkeit fiskalpolitischer Maßnahmen in Krisenzeiten bestätigt sich auch in empirischen Analysen (Auerbach und Gorodnichenko, 2012). Dies gilt insbesondere für öffentliche Investitionen, deren Effektivität in Krisenzeiten besonders hoch ist (Gechert und Rannenberg, 2018).

## Schwerpunktsetzung verbesserungsfähig

Das von der Bundesregierung auf den Weg gebrachte Konjunkturpaket ist demnach grundsätzlich sinnvoll und mit einem Volumen von 3,8 % des Vorkrisen-BIP in den Jahren 2020 und 2021 auch zu einem kräftigen Anschub fähig. Hinsichtlich der konjunkturellen Wirksamkeit wesentlicher Elemente liefert die jüngere Multiplikatordebatte einige Erkenntnisse. Insgesamt haben öffentliche Investitionen einen besonders starken Effekt auf das Wachstum, während steuerliche Maßnahmen einen eher niedrigen kurzfristigen Multiplikator aufweisen, der auch weniger sensitiv auf das makroökonomische Umfeld reagiert (Gechert und Rannenberg, 2018). Bei den potenziell konsumstimulierenden Maßnahmen schneidet tendenziell eine Ausweitung von Transfers deutlich besser ab als Steuersenkungen. Gechert et al. (2020) vergleichen die Multiplikatoreffekte zusätzlicher Sozialausgaben mit einer Senkung der Sozialversicherungsbeiträge in Deutschland. Wenn man Einnahmen- und Ausgabenschocks miteinander vergleicht, zeigt sich, dass die Ausgaben einen stärkeren positiven Effekt auf das BIP haben, der auch persistenter ist und mit höherer Genauigkeit geschätzt wird. Der Fiskalmultiplikator für Sozialausgaben beträgt etwa 1,1 zum Zeitpunkt des Schocks, während er bei den Einnahmen nur 0,4 beträgt. Die ermittelten durchschnittlichen Multiplikatoreffekte können als erster Anhaltspunkt auch für Maßnahmen dienen, die in der gegenwärtigen Krise umgesetzt werden. Vermutlich wäre die Wirkung sogar noch größer, denn die Evidenz spricht recht klar dafür (Gechert et al., 2015), dass finanzpolitische Maßnahmen in Krisenzeiten deutlich wirksamer sind. Gezielte Transfers scheinen in solchen Zeiträumen besonders effektiv zu sein (Bayer et al., 2020).

Steuersenkungen sind daher als Konjunkturimpuls weniger geeignet. Insofern ist es nicht optimal, dass der Fokus des Konjunkturpakets bezüglich der Unterstützung des privaten Konsums auf der bis zum 31.12.2020 befristeten Senkung der Umsatzsteuer liegt. Auch angesichts der Überwälzungsproblematik ist es durchaus ungewiss, ob diese Maßnahme in der gewünschten Stärke auf den privaten Konsum durchschlagen wird (Dullien und Gechert, 2020). Effizienter ließe sich der Konsum stimulieren, wenn die Finanzmittel in Richtung der Empfänger von Sozialleistungen, zum Teil die ohnehin benachteiligten und schwächeren Personen der Gesellschaft, gelenkt werden. Dies wäre auch wirksamer, um die aus der aktuellen Krise resultierende Zunahme der Armut abzuschwächen. Für Deutschland würde sich im Zuge der Coronakrise beispielsweise eine temporäre Aufstockung des Kurzarbeitergeldes gerade für kleinere Einkommen, ein höheres und länger gezahltes Arbeitslosengeld I, sowie direkte Hilfen für Minijobber anbieten, denen derzeit lediglich das Arbeitslosengeld II zur Verfügung steht. Ebenfalls sinnvoll wäre eine deutliche Aufstockung des Kinderbonus, der sehr effektiv Haushalte mit kleinen und mittleren Einkommen entlastet, aber mit nur 300 Euro je Kind gering dimensioniert ist.

Sehr zu begrüßen sind die investiven Elemente des Konjunkturpakets. Neben der potenziell hohen Multiplikatorwirkung dürfte von ihnen auch ein dauerhafter und teilweise transformativer Effekt in den Bereichen Klimaschutz und Digitalisierung ausgehen. Allerdings hätte der investive und transformative Aspekt durchaus stärker betont werden können; hierzu gab es zahlreiche Vorschläge (Bach et al., 2020).

## Zusätzliche Unterstützung für Kommunen erforderlich

Ein unter investiven Gesichtspunkten sehr wesentlicher Aspekt ist außerdem, dass die Unterstützung der Kommunen im Konjunkturpaket zu gering ausgeprägt ist: Die Kommunen sind wesentlich für die öffentliche Daseinsvorsorge und tätigen mehr als ein Drittel der öffentlichen

Investitionen. Bund und Länder ersetzen die erwarteten Gewerbesteuerausfälle für 2020, und der Bund stockt dauerhaft seinen Anteil an den Kosten der Unterkunft in der Grundsicherung von 50 % auf bis zu 75 % sowie bestehende Investitionsförderungen auf.

Insgesamt erhalten die Kommunen damit in den Jahren 2020 und 2021 über 20 Mrd. Euro an Unterstützung. Dies wird jedoch nicht ausreichen, denn die Belastungen der Kommunen durch die Coronakrise sind weitaus größer. Laut Steuerschätzung vom Mai 2020 werden sich die Steuerausfälle bis 2024 auf über 45 Mrd. Euro summieren. Hinzu kommen kommunale Haushaltsbelastungen aus verschiedenen steuerlichen Maßnahmen: Die steuerlichen Maßnahmen des Konjunkturpakets liegen kumuliert bis zum Jahr 2024 bei etwa 9 Mrd. Euro. Des Weiteren ergeben sich Mindereinnahmen aus dem jüngsten Entwurf des Zweiten Gesetzes zur steuerlichen Entlastung von Familien in Höhe von nochmals 6 Mrd. Euro. Über den Kommunalen Finanzausgleich werden Städte und Gemeinden in fast allen Bundesländern automatisch an den krisen- und steuerreformbedingten Steuerausfällen der Länder, die in den Steuerverbund eingehen, beteiligt. Daraus könnte insgesamt eine weitere Einnahmeminderung von 23 Mrd. Euro resultieren.

Damit drohen bei den derzeitigen Konjunkturaussichten bis 2024 massive Nettobelastungen der Kommunen in einer Größenordnung von 60 Mrd. Euro. Die Kommunen brauchen also dringend weitere Unterstützung durch Bund und/oder Länder. Wenn sich dafür im Bund keine Mehrheit finden lässt, müssen die Länder im Rahmen des kommunalen Finanzausgleichs die Einnahmenausfälle kompensieren und gegebenenfalls Entschuldungsfonds auflegen, um auch überschuldeten Kommunen endlich wieder eine Investitionsperspektive zu eröffnen.

### Schuldenbremse als Risiko für Aufschwung und Investitionen

Für die weitere konjunkturelle Erholung und die mittelfristigen Wachstumsperspektiven ist es von zentraler Bedeutung, die Normalisierung der Finanzpolitik in der Phase der Konjunkturerholung vorsichtig sowie konjunktur- und investitionsgerecht zu gestalten. Die Erfahrungen mit der Austeritätspolitik während der Eurokrise zeigen, dass verfrühte Konsolidierungsmaßnahmen zu stark negativen Effekten auf die Wirtschaftsleistung führen (Blanchard und Leigh, 2013; House et al., 2019), die langfristig aufgrund von Hysterese-Effekten auch das Produktionspotenzial senken (Fatás und Summers, 2018; Gechert et al., 2019). Die schnelle Haushaltskonsolidierung nach der globalen Finanz- und Wirtschaftskrise in Deutschland seit 2010 erfolgte auch nicht durch forcierte Kürzungen, sondern größtenteils durch das Auslaufen konjunktureller Impulse und die überraschend schnelle und starke konjunkturelle Erholung (Rietzler und Truger, 2019). Daher sind diskretionäre Ausgabenkürzungen und/oder Steuer- und Abgabenerhöhungen zu vermeiden. Damals wie heute waren und sind dauerhafte diskretionäre Steuersenkungen ebenfalls nicht angebracht. Sie reißen strukturelle Löcher in die Haushalte und führen zu unnötigem ausgabeseitigen Konsolidierungsdruck. Gerade die Corona-Krise sollte die Bedeutung eines finanziell gut ausgestatteten und handlungsfähigen Staates eindringlich vor Augen geführt haben.

Daher sollte die Ausnahmeregel der Schuldenbremse in Bund und Ländern so lange wie notwendig angewendet werden. Trotz dieser Möglichkeit birgt die Schuldenbremse in ihrer gegenwärtigen Ausgestaltung erhebliche Risiken für die wirtschaftliche Erholung. Die Methode der EU-Kommission, die im Rahmen der Schuldenbremse des Bundes und vieler Bundesländer zur Anwendung kommt, hat sich als problematisch erwiesen, weil das von ihr errechnete Produktionspotenzial stark von der jeweils aktuellen Konjunkturlage beeinflusst wird. So wird das Produktionspotenzial in Abschwungphasen schnell und stark nach unten revidiert, während es in Aufschwungphasen entsprechend schnell nach oben gezogen wird. Dass die Berechnung des Produktionspotenzials so konjunkturanfällig ist, hat sehr konkrete und gravierende Folgen für die errechneten strukturellen Haushaltssalden: Die öffentlichen Haushalte und besonders die Steuereinnahmen reagieren auf die tatsächliche wirtschaftliche Situation, also im Falle einer Abschwächung auf den tatsächlichen Rückgang des BIP im Vergleich zur Planung. Die Konjunkturbereinigung stuft dann jedoch regelmäßig nur einen Teil dieser tatsächlichen Reaktion als konjunkturell ein.

Mittlerweile lässt sich die Dramatik dieses Effekts am Beispiel der jüngsten Revision der gesamtwirtschaftlichen Projektion der Bundesregierung illustrieren (Heimberger und Truger, 2020). So wurde das Produktionspotenzial für das Jahr 2021 von der Herbstprojektion 2019 zur Frühjahrsprojektion 2020 um 2,4 % real und 2,9 % nominal abwärts revidiert. In den für die Finanzplanung des Bundes und der Länderhaushalte relevanten Jahren 2022 bis 2024 wird die Abweichung immer größer und steigt auf 3,0 % real bzw. 3,7 % nominal an.

Das hat zur Folge, dass sich der strukturelle gesamtstaatliche Budgetsaldo im Jahr 2021 aufgrund der Potenzialrevision um 1,6 Prozentpunkte verschlechtert; im Jahr 2024 sind es dann 2,1 Prozentpunkte. Allein für den Bund beträgt die revisionsbedingte Verschlechterung 2021 0,6 % des BIP oder gut 20 Mrd. Euro, im Jahr 2024 sind es dann schon 0,9 % des BIP. Falls diese Potenzialrevisionen in der Haushaltsplanung von Bund und den betroffenen Ländern tatsächlich wirksam würden, müsste die Finanzpolitik schon 2021 restriktiver ausgestaltet werden als angemessen. Zudem könnte es aufgrund der relativ klein angesetzten Produktionslücken schwieriger werden 2021 oder in den Folgejahren erneut die Ausnahmeregel der Schuldenbremse in Anspruch zu nehmen.

Hinzu kommen die Tilgungsverpflichtungen, die Bund und Länder für die im Rahmen der Ausnahmeregel der Schuldenbremse aufgenommenen zusätzlichen Schulden eingegangen sind. Wird die Konjunkturbereinigung nicht angepasst und werden die in einigen Fällen kurzen Tilgungszeiträume von unter zehn Jahren nicht deutlich gestreckt, droht der deutschen Finanzpolitik perspektivisch ein drastischer ökonomisch schädlicher Konsolidierungskurs. Aus diesem Grund sollte kurzfristig auf Potenzialrevisionen verzichtet oder aber der Potenzialpfad statistisch deutlich geglättet werden. Zudem sollten existierende Rücklagen geschont und die Tilgungspläne für im Rahmen der Ausnahmeregel der Schuldenbremse aufgenommene Kredite gestreckt und konjunkturgerecht ausgestaltet werden.

Mittelfristig steht die deutsche Finanzpolitik vor der Aufgabe, den für die anstehende klimapolitische und digitale Transformation notwendigen massiven Bedarf an öffentlichen Investitionen zu finanzieren (Bardt et al., 2019). Hierfür muss die Nutzung existierender Spielräume im Rahmen von Schuldenbremse und europäischem Fiskalregelwerk, aber auch die konjunktur- und investitionsgerechte Reform der Schuldenbremse ganz nach oben auf die finanzpolitische Prioritätenliste (Bardt et al., 2019; Schnabel und Truger, 2020).



Literatur

Auerbach A J Gorodnichenko Y Measuring the output responses to fiscal policy American Economic Journal: Economic Policy 2012 4 2 1 27
Bach, S., H. Bär, K. Bohnenberger, S. Dullien, C. Kemfert, M. Rehm, K. Rietzler, M. Runkel, S. Schmalz, S. Tober und A. Truger (2020), Sozialökologisch ausgerichtete Konjunkturpolitik in und nach der Corona-Krise. Forschungsvorhaben im Auftrag des Bundesministeriums für Umwelt, Naturschutz und nukleare Sicherheit, IMK Study, 68, Mai.
Bardt, H., S. Dullien, M. Hüther und K. Rietzler (2019), Für eine solide Finanzpolitik: Investitionen ermöglichen!, IMK Report, 152 bzw. IW-Policy Paper, 10/19, November, Düsseldorf und Köln.
Bayer C., B. Born, R. Luetticke und G. J. Müller (2020), The Coronavirus Stimulus Package: How large is the transfer multiplier?, CEPR Discussion Paper, DP14600.
Blanchard, O. J. und D. Leigh (2013), Growth Forecast Errors and Fiscal Multipliers, NBER Working Paper, 18779.
Christiano L Eichenbaum M Rebelo S When is the government spending multiplier large? Journal of Political Economy 2011 119 78 121 10.1086/659312
Dullien, S. und S. Gechert (2020), Wie wird sich die temporäre Umsatzsteuer-Senkung auswirken?, Makronom Blog, 10. Juni, https://mak-ronom.de/konjunkturpaket-wie-wird-sich-die-temporaere-umsatz-steuer-senkung-auswirken-36241 (8. Juli 2020).
Elmendorf, D. W. und J. Furman (2008), Three Keys to Effective Fiscal Stimulus, Brookings Institution, 26. Januar, https://www.brookings.edu/opinions/three-keys-to-effective-fiscal-stimulus/ (8. Juli 2020).
Fatäs A Summers L H The permanent effects of fiscal consolidations Journal of International Economics 2018 112 238 250 10.1016/j.jinteco.2017.11.007
Furman, J. (2016), The New View of Fiscal Policy and Its Application, Delivery for Conference: Global Implications of Europe’s Redesign, 5. Oktober, New York, https://obamawhitehouse.archives.gov/sites/default/files/page/files/20161005_furman_suerf_fiscal_policy_cea.pdf (8. Juli 2020).
Gechert S Horn G Paetz C Long-Term Effects of Fiscal Stimulus and Austerity in Europe Oxford Bulletin of Economics and Statistics 2019 81 3 647 666 10.1111/obes.12287
Gechert, S., A. Hughes Hallet und A. Rannenberg (2015), Fiscal multipliers in downturns and the effects of Eurozone consolidation, voxeu Blog, 26. Februar, https://voxeu.org/article/fiscal-multipliers-and-eurozone-consolidation (8. Juli 2020).
Gechert, S., C. Paetz und P. Villanueva (2020), The Macroeconomic Effects of Social Security Contributions and Benefits, Journal of Monetary Economics, (im Erscheinen).
Gechert S Rannenberg A Which Fiscal Multipliers Are Regime-Dependent? A Meta-Regression Analysis Journal of Economic Surveys 2018 32 4 1160 1182 10.1111/joes.12241
Heimberger, P. und A. Truger (2020), Der Outputlücken-Nonsens gefährdet Deutschlands Erholung von der Corona-Krise, Makronom, 2. Juni, https://makronom.de/der-outputluecken-nonsense-gefaehrdet-deutschlands-erholung-von-der-corona-krise-36125 (8. Juli 2020).
House, C., C. Proebsting und L. Tesar (2019), Austerity in the aftermath of the great recession, Journal of Monetary Economics, (im Erscheinen).
Rietzler K Truger A Is the „Debt Brake“ behind Germany’s successful fiscal consolidation? Revue de l’OFCE, Supp. 2019 2 6 11 30
Schnabel, I. und A. Truger (2020), Eine andere Meinung, Minderheitsvotum zu Kapitel 5 „Die Schuldenbremse: Nachhaltig, stabilisierend, flexibel, in Sachverständigenrat zur Begutachtung der gesamtwirtschaftlichen Entwicklung (2020), Den Strukturwandel meistern, Jahresgutachten 2019/20, Wiesbaden, 298–304.
Woodford M Simple analytics of the government expenditure multiplier American Economic Journal: Macroeconomics 2011 3 1 35
